# Effect of Semelil, an Herbal Selenium-Based Medicine, on New Bone Formation in Calvarium of Rabbits

**DOI:** 10.1155/2018/2860367

**Published:** 2018-02-26

**Authors:** Amir Alireza Rasouli-Ghahroudi, Amirreza Rokn, Mohammad Abdollahi, Fatemeh Mashhadi-Abbas, Siamak Yaghobee

**Affiliations:** ^1^Department of Periodontics and Dental Implant Research Center, School of Dentistry, Tehran University of Medical Sciences, Tehran, Iran; ^2^Toxicology and Disease Group, Pharmaceutical Sciences Research Center and Department of Toxicology and Pharmacology, Faculty of Pharmacy, Tehran University of Medical Sciences, Tehran, Iran; ^3^Oral Maxillofacial Pathology Department, School of Dentistry, Shahid Beheshti University of Medical Sciences, Tehran, Iran

## Abstract

**Background:**

This study aims to analyze the effect of Semelil, an herbal selenium-based medicine, on osteogenesis in rabbit calvarium defects.

**Methods:**

Four identical bony defects (8 mm) were created in the calvarium of 16 New Zealand male rabbits and filled randomly with xenogenic bone substitute material (Bio-Oss®) and semelil herbal drug (ANGIPARS™). One site was filled with Bio-Oss (B); the second site was treated with ANGIPARS (A); the third site was treated with ANGIPARS + Bio-Oss (AB); and the fourth site was left as untreated control (C) and defects were left unfilled. Rabbits were randomly divided into two groups (*n* = 8) and sacrificed at four and eight weeks. Percentage of new bone formation, type of the newly formed bone, percentage of the remaining xenograft biomaterial, and foreign body reaction (FBR) were evaluated via histological and histomorphometric analyses.

**Results:**

The percentage of new bone formation was significantly different among four groups. The highest effect was observed in AB, followed by A, B, and C groups, respectively. The difference in the mean percentage of new bone formation between four and eight weeks was significant for all four groups (*P* < 0.001). Regarding bone formation, the interaction effect of A and B was significant at four (*P* < 0.001) and eight weeks (*P* = 0.002). ANGIPARS alone and in presence of Bio-Oss enhanced new bone formation at both four and eight weeks (*P* < 0.001). The mean amount of new bone formation was significantly different at four and eight weeks in groups C (*P* = 0.008), A (*P* < 0.001), B (*P* < 0.001), and AB (*P* = 0.003). FBR was not observed in any group.

**Conclusion:**

Semelil may be useful as an adjunct to conventional osteoconductive materials in order to enhance osteogenesis.

## 1. Introduction

Bone graft materials have extensive clinical applications in medicine and dentistry [1]. Most manufacturers claim to produce bone grafts with suitable physical, chemical, and biological properties [2]. Clinicians have always been in search for high-standard biomaterials to achieve a regenerative and reconstructive procedure. Autogenous bone is often referred as the gold standard for regenerative and reconstructive procedures due to its optimal biological properties. Autogenous bone grafts (ABGs) may be procured from the iliac crest, mandibular symphysis, ribs, and tibia orcalvarium [3]. The biological mechanisms involved in new bone formation at reconstructed sites include osteoinduction, osteoconduction, and osteogenesis [4, 5]. Autogenoueos bone has all of these characteristics. In most cases, all the three mechanisms are involved in the process of bone regeneration. In fact, osteogenesis does not occur completely in the absence of osteoconductive and osteoinductive mechanisms. In other words, simultaneous presence of the three following requirements is necessary to achieve osteogenesis: (A) presence of osteoblasts or cells with the potential for differentiation into bone forming cells; (B) presence of osteoinductive stimuli to initiate the differentiation of mesenchymal cells to osteoblasts; and (C) presence of an osteoconductive environment to form a scaffold for growth and proliferation of preosteoblastic cells and their differentiation into osteoblasts for new bone formation. Autogenous bone has some disadvantages and complications specially unpredictable outcome, postoperative infection, inadequate quantity, the need for an extra donor site, patient discomfort, donor site morbidity, and graft resorption [4–6]. A biomaterial possessing the three aforementioned properties would be most ideal.

To overcome these limitations, alternative biomaterials have been developed. Bone graft materials available in the market are mostly osteoconductive rather than osteoinductive or osteogenic. To date, most previous studies conducted on bovine bone products like Bio-Oss have shown that this graft material is biocompatible and mainly osteoconductive. Bio-Oss is inorganic bovine bone mineral and is known as a gold standard bone substitute. Its organic content has been chemically removed and thus can be used in hosts after sterilization [7, 8].

Semelil (ANGIPARS) is a recently marketed herbal medicine produced from the extract of* Melilotus Officinalis* (yellow sweet clover), which belongs to the Fabaceae Legume Family. ANGIPARS has been recently introduced as an effective medicine for treatment of diabetic foot ulcer [9]. It facilitates wound healing, improves the quality of the repair tissue at the wound site, and decreases the rate of recurrence [10]. This product has minimal toxicity and not only decreases wound size but also improves microvascularization of tissues [11, 12]. It also contains variable amounts of selenium, urea, fructose, sodium phosphoglycerol, 7-hydroxycoumarine, and flavonoids, which have potent antioxidant and neuroprotective properties. Since angiogenesis is a key factor in bone formation and the main mechanism of action of ANGIPARS is via angiogenesis and increasing the tissue blood flow and oxygenation [8], we hypothesized that ANGIPARS may be able to enhance osteogenesis. Therefore, the present study was undertaken to assess the efficacy of ANGIPARS in combination with or without Bio-Oss in new bone formation at the site of bone defects in rabbit calvarium.

## 2. Materials and Methods

This experimental animal study was approved by the Ethics Committee of Tehran University of Medical Sciences with the code number of 91-03-69-14897. Maintenance and care of experimental animals complied with the internationally accepted guidelines for the care and use of laboratory animals. Sixteen healthy white New Zealand male rabbits with a mean weight of 2.5 kg were used. Of 16, 8 were sacrificed at four weeks and the rest at 8 weeks. The sample size was calculated according to the previous study by Rokn et al. [13] considering 80% power and 95% confidence interval (CI). Animals were kept in the animal room of the Faculty of Pharmacy in separate cages on a uniform standard feeding regimen for two weeks prior to the experiment. Semelil (ANGIPARS) was generously delivered by the ParsRoos Co. (Tehran, Iran). Bio-Oss bone substitute (0.25 mm–1 mm granules) was purchased from Geislitch Pharma AG (Wolhusen, Switzerland).

### 2.1. Surgical Procedure

**Figure 1:**
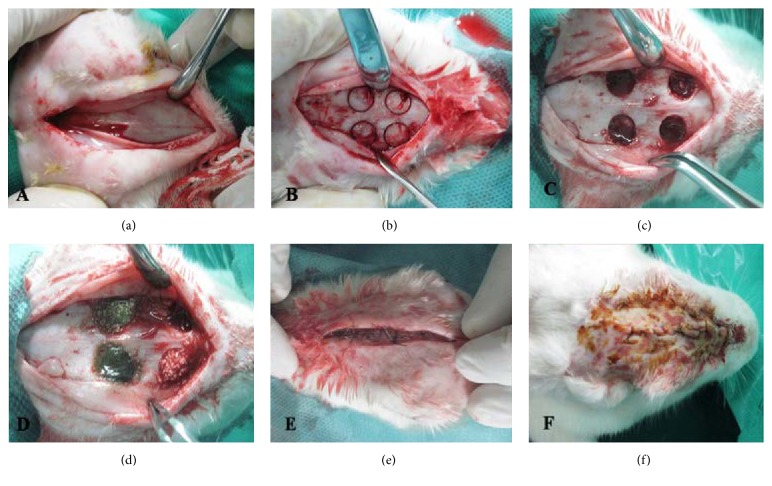
*Surgical Procedure*. (a) 10 cm craniocaudal incision was made and a mucoperiosteal flap was elevated (b, c). Four identical circular defects (8 mm in diameter, two in the frontal and two in the parietal bone) were created in the calvarium using a trephine bur (d). The defects were filled with ANGIPARS, ANGIPARS plus Bio-Oss, and Bio-Oss alone and the other one is left empty (e, f). The periosteum and the calvarial skin were then sutured with 4.0 vicryl (Ethicon, Somerville, NJ, USA) and 3.0 nylon (Ethicon, Somerville, NJ, USA) sutures, respectively.

Animals were anesthetized via intramuscular injection of 2% xylazine and 10% ketamine. The calvarium was scrubbed with 7% Betadine for 5 minutes and the fur on the surgical site was shaved. After preparation and draping, the area was scrubbed with 7% Betadine for 5 minutes. Using #15 scalpel, a 10 cm craniocaudal incision was made and a mucoperiosteal flap was elevated using a periosteal elevator (Figure 1(a)). To standardize the location of defects, anatomical landmarks were used, including the occipital process and the craniocaudal suture. Using a low-speed hand piece and a trephine bur with an internal diameter of 7 mm and an external diameter of 8 mm, four identical circular defects (two in the frontal and two in the parietal bone) were created in the calvarium under copious saline irrigation (Figures 1(b) and 1(c)). Three types of materials were used to fill three of the defects: (1) ANGIPARS (A), (2) Bio-Oss with a particle size of 250 to 1000 *μ*m (B), and (3) ANGIPARS plus Bio-Oss (AB). The fourth defect was left unfilled and considered as the control. To eliminate bias in defect location, the sequence of the filling of the defects, two in frontal bone and two in parietal bone, was varied as follows. In the first rabbit, the defects were treated randomly with the three aforementioned materials and the fourth defect was left unfilled as the control. Then these positions were changed rotationally (clockwise) for the other rabbits (Figure 1(d)). All locations were recorded on charts. Following placement of the materials, the periosteum and the calvarial skin were then sutured with 4.0 vicryl (Ethicon, Somerville, NJ, USA) and 3.0 nylon (Ethicon, Somerville, NJ, USA), respectively (Figures 1(e) and 1(f)). Animals were transferred to a room with 37°C temperature for recovery and 0.1 ml of ketoprofen was administered daily for three days to control pain and swelling. 0.6 ml of enrofloxacin (Baytril, Bayer Corp., Shawnee, KS, USA) was also administered subcutaneously for 5 days. Of the 16 rabbits that were operated, eight (group 1) were sacrificed randomly after four and the remaining eight (group 2) after eight weeks by injection of 2 mL sodium thiopental into their marginal auricular venules. The calvarium was resected using a saw and fixed in 10% buffered formalin for histological analysis (Figure 1).

### 2.2. Laboratory Phases

Specimens were separately stored in 10% formalin for two weeks for complete fixation and were then immersed in 10% nitric acid solution for one week. They were evaluated daily during this time period to control decalcification. Specimens were then immersed in 20% lithium carbonate for neutralization. The defects were then sectioned from their longest diameter horizontally and coded by a three-digit code. The left digit indicated the evaluation time point (4 or 8 weeks), the middle digit indicated the respective rabbit (number 1 to 8), and the right digit indicated the respective defect [AB (ANGIPARS + Bio-Oss), A (ANGIPARS), B (Bio-Oss), and C (control)]. Paraffin embedded blocks were routinely prepared; 3 *μ*m slices were sectioned for hematoxyline and eosin (H & E) staining and further histolgical/histomorphometric analyses.

### 2.3. Histological and Histomorphometric Analyses

Specimens were observed under a light microscope (BX-41, Olympus, Japan) by a pathologist blinded to the coding of specimens. The following parameters were evaluated in the specimens and scored.

#### 2.3.1. Foreign Body Reaction (FBR)

This phenomenon was determined based on the presence of giant cells and granulomatous reaction at ×40 magnification. Presence of FBR was categorized as follows:

  Score 0: 0 foci
  Score 1: 0–10 foci
  Score 2: 10–20 foci
  Score 3: More than 20 foci

#### 2.3.2. Bone Vitality

This parameter was determined based on the presence of osteocytes inside trabecular bone lacunae at ×40 magnification and categorized as vital or non-vital.

#### 2.3.3. Type of Newly Formed Bone (NFB)

Order and orientation of collagen fibers in the NFB were evaluated under polarized light at ×40 magnification. A type of NFB was categorized as follows:

  Score I: woven bone alone
  Score II: both lamellar and woven bone
  Score III: lamellar bone alone
  Score IV: osteoid formation

#### 2.3.4. Percentage of NBF

This measurement defines the percentage of the entire defect occupied by the NFB. To calculate the percentage of new bone formation, histological sections were photographed at ×40 magnification via a digital camera (E8400, Nikon, Japan). Using Iranian histomorphometric software version 1 (SBMU, Tehran, Iran), the mean percentage of areas occupied by bone was calculated.

#### 2.3.5. Percentage of Remaining Biomaterial

This defines the percentage of total defect occupied by Bio-Oss particles. To calculate this variable, digital photographs were obtained of H&E stained histological slides at ×40 magnification and the mean percentage of areas occupied by the biomaterial was calculated using the Iranian histomorphometric analysis software version 1.

#### 2.3.6. Location of Osteogenesis in Defects

  Grade 1: central
  Grade 2: marginal
  Grade 3: central and marginal

### 2.4. Statistical Analysis

The quantitative variables were reported as mean and standard deviation and as raw number and percentage. To compare qualitative data among the four groups (A, B, AB, and C), Fisher's Exact test was used. To analyze the effect of application of Bio-Oss and ANGIPARS on quantitative variables, including new bone formation, two-way ANOVA was used. Student's *t*-test was applied when the interaction effect of the two factors was significant. The distribution of percentage of the remaining biomaterial was not normal. Thus, the effect of the two factors was analyzed using Mann Whitney *U* test, separately. *P* < 0.05 was considered statistically significant.

## 3. Results

During the postoperative period, none of the animals were lost. Eight of the rabbits were sacrificed randomly at four weeks and the remaining eight at eight weeks, 16 Bio-Oss, 16 ANGIPARS plus Bio-Oss, 16 ANGIPARS, and 16 empty defects were evaluated. The FBR was not observed in any specimen. Giant cells or granulomatous reaction were not seen in any defect. All the NFB in defects was vital.

**Figure 2:**
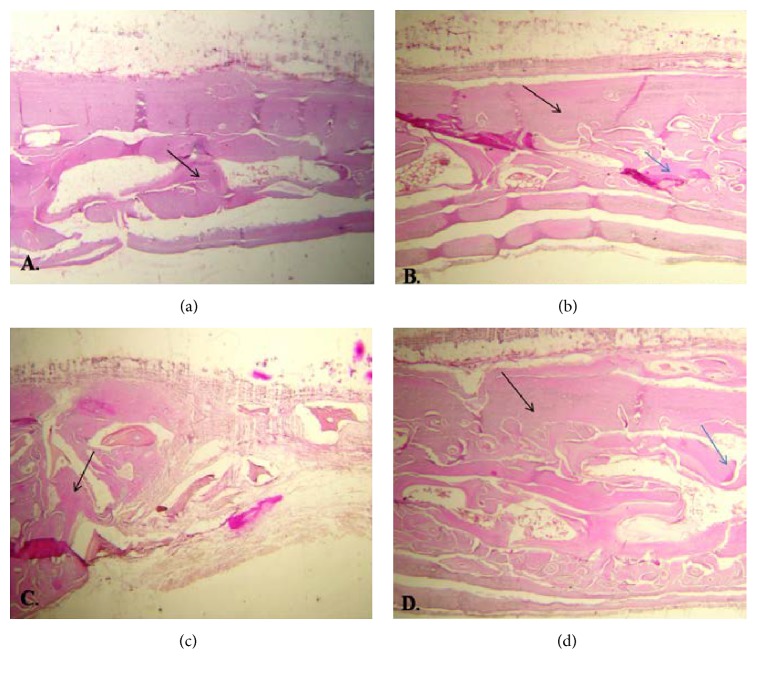
Bone formation at 4 weeks in samples at ×40 magnification (black arrow = regenerated bone; blue arrow = remaining grafting material). (a) Control, (b) Bio-Oss sample, (c) ANGIPARS, and (d) ANGIPARS plus Bio-Oss. As shown, the most trabecular bone pattern was seen in ANGIPARS plus Bio-Oss group.

**Figure 3:**
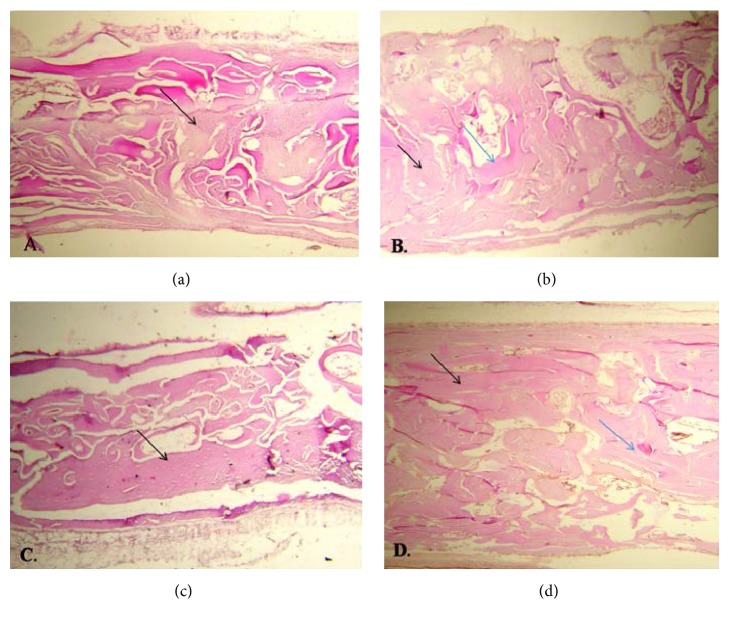
Bone formation at 8 weeks in samples at ×40 magnification (black arrow = regenerated bone; blue arrow = remaining grafting material). (a) Control, (b) Bio-Oss sample, (c) ANGIPARS, and (d) ANGIPARS plus Bio-Oss. As observed, the most trabecular bone pattern was seen in ANGIPARS plus Bio-Oss group.

At four weeks, lamellar bone was not seen in any defect while woven-lamellar bone was only seen in A and AB groups (Figure 2). At eight weeks, all defects in AB group were of lamellar bone (Figure 3) (Table 1). According to Fisher's exact test, the difference in the type of the NFBat four and eight weeks was significant in the control (*P* = 0.001), Bio-Oss (*P* < 0.001), ANGIPARS (*P* = 0.02), and ANGIPARS + Bio-Oss (*P* < 0.001) groups.

The percentage of total NBF is demonstrated in Table 3. According to two-way ANOVA, the interaction effects of materials in defects are shown in Table 3.

Based on *t*-test, the mean percentage of NBF between four and eight-week time points was significant in the control (*P* < 0.001), B (*P* < 0.001), A (*P* < 0.001), and A + B (*P* < 0.001) groups.

### 3.1. Percentage of Remaining Biomaterial

At four weeks, in group B, the mean, maximum, and minimum percentages of remaining Bio-Oss biomaterials were 23.15, 26.75, and 6.90, respectively. At eight weeks, these rates were 4.16, 10.58, and 1.05, respectively. In group AB, the mean, maximum, and minimum percentage of remaining BioOss were 23.40, 28.33, and 4.44, respectively, at four weeks and 7.29, 9.87, and 2.13, respectively, at eight weeks. The change was statistically significant at four (*P* < 0.001) and eight (*P* < 0.001) weeks.

### 3.2. Location of Bone Formation in the Defects

Based on Fisher's exact test, the difference in location of bone formation was significant at four (*P* = 0.008) but not significant at eight weeks (*P* = 1.00) among groups. At four weeks, four specimens in group C only demonstrated marginal bone formation. The remaining samples showed bone formation at the center and the margins. At eight weeks, only one specimen in group C did not show bone formation at the center. The remaining specimens demonstrated bone formation at the center and the margins.

## 4. Discussion

Many techniques have been recently introduced to enhance bone regeneration [14]. A bone substitute is necessarily required to enhance bone regeneration and healing in bony defects. Autogenous bone graft as the gold standard technique is mainly associated with donor site morbidity, graft resorption, limited quantity, and patient discomfort [15, 16]. One solution is to use bone substitute materials alone as an osteoconductive scaffold for regeneration of bone filling defect [17]. Thus, demand for nonautogenous bone grafts is increasing due to availability, ease of storage, and sterility [18–20].

Our study is the first animal study to assess the efficacy of Semelil (ANGIPARS) in osteogenesis alone and in combination with Bio-Oss, a popular bone graft substitute, in order to assess if this material can promote bone formation. Systemic application of ANGIPARS has no adverse effect on bone formation [21]. Results of an animal study showed that, during the study period, no significant difference existed in biochemical or hematologic parameters between the test and control groups. Evidence shows that ANGIPARS is well tolerated and has no adverse effects on the function of body organs in subacute and chronic toxicity tests [22].

In the current study, the efficacy of ANGIPARS with and without Bio-Oss for bone regeneration in experimentally created defects was compared in rabbit calvarium. Rabbit cranial defects are the first choice as a bone model for bone grafting and bone regeneration studies because they provide adequate bone marrow that facilitates bone formation [23, 24]. The cranium of a rabbit is larger than that of rats and a greater number of defects can be created in a rabbit calvarium. Therefore, the duration of surgery, the costs, and the visual errors can be decreased. The bone remodeling phase occurs three times faster in rabbits than in humans [24–28]. Thus, two- to four-week recovery period is considered adequate for evaluation of the early response. Eight weeks or longer time intervals can be considered for evaluation of the delayed phase of healing, that is, suture closure, biomaterial resorption, bone remodeling, or rate of bone regeneration [24–28].

Although, in the experimental defects, osteogenesis was noted in the 8 mm control defects, it was significantly less than that of other groups. This finding may be due to the suturing of the thick periosteum of the rabbit calvarium over the experimental defects, mimicking the effect of guided bone regeneration (GBR). Bone regeneration is the basis of GBR. In this process, the blood clot is stabilized, defect space is maintained, and the surgical site does not undergo mechanical loading [29, 30]. Accordingly, NBF was significantly higher in groups A, B, and AB at four and eight weeks than that of group C. This demonstrated the positive effect of experimental materials used in our study. Thus, we believe that creating four circular defects (8 mm in diameter) in rabbit calvarium provides a suitable model for evaluation of the healing phase and comparison of the efficacy of different materials simultaneously in order to decrease individual variations in experimental studies. Results obtained in such conditions can better be generalized to human periodontal lesions because, in the clinical setting, periodontal bone defects around teeth usually have dimensions smaller than 8 mm [31–33].

In the current study, a significant difference was observed regarding the overall percentage of NBF at both four and eight weeks in the following order from the highest to the lowest: AB > A > B > C. However, assessment of NBF at the center of defects revealed the following order: AB > A > B > C and A > AB > B > C at four and eight weeks, respectively.

Previous studies have demonstrated that Bio-Oss is a osteoconductive biocompatible graft material that can promote bone formation [13, 31, 32]. Lindhe et al. demonstrated that percentage of Bio-Oss particles decreased over time indicating their replacement with host bone in the long term [20]. In the current study, NBF in the Bio-Oss group was significantly higher than in the control group, which is in accordance with the aforementioned studies. However, there are some studies reporting that Bio-Oss is resistant to resorption and its particles may remain in the graft site up to four years [32]. Khorsand et al. found no significant difference in this regard between the Bio-Oss and control groups at four, six, and eight weeks [34]. In the current study, ANGIPARS alone significantly increased NBF in both the center and margins of defects compared to the Bio-Oss and control groups. Melilotus, coumarin, and flavonoids are the main constituents of ANGIPARS. It has been demonstrated that coumarin and vitamin K products increase osteogenic markers, that is, osteocalcin and alkaline phosphatase (ALP), and probably decrease urinary calcium and secretion of hydroxy proline (bone loss markers) [21].

Tang et al. demonstrated that a coumarin derivative stimulates NBF following local subcutaneous injection into the calvarium and could increase the biomechanical bone strength. It also increased osteoblastic differentiation under in vitro conditions via the bone morphogenetic protein-2 (BMP-2) and Wnt/*β*-catenin signaling pathways. BMP-2 bonds to receptors on the surface of bone cells and phosphorylates SMAD 1/5/8; as a result, some specific bone genes are expressed [35]. Ostholeinhibits bone resorption by its estrogen-like effects on ovariectomized rats, facilitates osteoid formation and mineralization [36, 37]. Chen et al. (2005) indicated the positive effects of flavonoids on improving bone strength, enhancing bone cell proliferation, and osteogenic differentiation of mesenchymal stem cells [38]. Xiao et al. (2014) demonstrated that flavonoids stimulated the proliferation and differentiation of preosteoblasts. Flavonoids significantly enhanced cell proliferation, activated ALP, and increased the expression of osteoprotegerin mRNA (OPG/RANKL) in mouse osteosarcoma cells [39].

Although a higher overall bone formation was found in the AB group during study. Assessment of the center of defects revealed a higher bone formation in this area in group A at the end of the course of study. These findings emphasize the direct effect of ANGIPARS on bone formation and its ability to enhance NBF when applied alone or in combination with Bio-Oss.

The authors believe that higher rate of osteogenesis in the ANGIPARS and ANGIPARS plus Bio-Oss groups may be attributed to the effect of 7-hydroxy coumarin and flavonoids as the main constituents of ANGIPARS. A possible mechanism is the increased expression of BMP-2 and its effect on proliferation and differentiation of preosteoblastic cells at the defect site. According to a study by Gao et al. (2013), appropriate concentrations of 7-methoxy-8-isopentyl coumarin not only increase cell proliferation but also induce the differentiation of periodontal ligament progenitor cells like the mesenchymal stem cells [40].

In our study, at both four and eight weeks, both ANGIPARS groups (A and/or AB) demonstrated higher formation of lamellar bone. This may be attributed to the presence of coumarins in ANGIPARS. Coumarins can induce the secretion of extracellular matrix, increase the uptake of calcium by the matrix, and enhance collagen type I secretion by increasing ALP activity in the cells, the matrix, and the formation of osteoblastic vesicles leading to mineralization [40] which could enhance the speed of bone maturation.

According to a study by Lin et al. (2014), BMP-2 enhances the proliferation and differentiation of mesenchymal stem cells and bone regeneration [41]. Since coumarins and flavonoids are the main constituents of ANGIPARS, they may induce osteogenesis via BMP-2, signaling pathways, which cause enhanced bone remodeling and maturation of defects filled with ANGIPARS. Studies have demonstrated that Bio-Oss is an osteoconductive bone substitute with the surface porosities that provide a suitable matrix for osteogenic cells that promote bone formation [13, 31, 32]. Thus, it is suggested that Bio-Oss combined with ANGIPARS can be a suitable composite graft material that not only increases the amount of NBF but also improves the quality and maturation of the NFB.

In this study, all the NFB in all defects was vital with no FBR. FBR or giant cells were not seen in the Bio-Oss defects; this is in line with some previous studies demonstrating that Bio-Oss is a biocompatible material that does not cause FBR [31, 32, 42, 43]. In contrast, other previous studies have shown presence of osteoclasts at the Bio-Oss graft area [42–44]. In a study by Rokn et al., all the defects in the Bio-Oss group showed mild FBR at four and eight weeks [33, 45]. Tapety et al. in a similar study on rabbits reported the presence of osteoclasts at 14 days in the Bio-Oss group [46]; this is not in line with the current study results. Literature review shows that flavonoids are capable of causing osteoclastic apoptosis and preventing bone loss [12, 47]. Moreover, coumarin has inhibitory effects on phagocytic activity and subsequent production of nitric oxide and metabolism by phagocytes [12]. Since ANGIPARS contains variable amounts of coumarin and flavonoid compounds, the authors believe that osteoclastic apoptosis caused by flavonoids and the inhibitory effects of coumarin can neutralize the FBR in ANGIPARS groups [8]. Besides, Bao et al. found that some of the ANGIPARS constituents can inhibit osteoclastogenesis by decreasing the resorption capacity of osteoclasts [48].

In our study, biomaterial remnants were assessed in the Bio-Oss groups (B and AB). This percentage was slightly (but not significantly) higher in the AB compared to the B group. A previous study demonstrated that* Magnolia Officinalis* decreased the number of leukocytes and polymorphonuclear cells in wounds and, subsequently, inhibited acute inflammation with its anti-inflammatory effect [12]. Considering the anti-inflammatory properties of ANGIPARS and consequent prevention of osteoclastic activity, this material can prevent Bio-Oss resorption; thus, the percentage of remaining biomaterials is expected to be higher in the AB group.

The current study showed that ANGIPARS not only promoted bone formation alone but also had a synergistic effect on both formation and maturation of new bone when applied along with Bio-Oss. It may be a suitable adjunct to confer osteogenic properties to materials like Bio-Oss, which are osteoconductive and space maintaining. Future human studies and clinical trials are required to assess the efficacy of ANGIPARS alone or as an adjunct to Bio-Oss and other conventionally used bone graft substitutes in the clinical setting.

## 5. Conclusion

Within the limitations of this animal study, ANGIPARS may be further examined as an adjunct to Bio-Oss for the purpose of enhancing bone healing and augmentation of bony defects.

## Figures and Tables

**Table 1:** Percentage and type of the NFB in examined defects.

Group	Time/NFB type					
	4 weeks			8 weeks		
	Lamellar	Woven	Woven-Lamellar	Lamellar	Woven	Woven-Lamellar
Control	0	100% (8)	0	75% (6)	12.5% (1)	12.5% (1)
Bio-Oss	0	100% (8)	0	75% (6)	0	12.5% (2)
AngiPars	0	0	100% (8)^a^	62.5% (5)	0	37.5% (3)
AngiPars + Bio-Oss	0	0	100% (8)^a^	100% (8)	0	0

^a^At four weeks in the ANGIPARS groups (alone or combined), lamellar bone formation was observed. The percentage of new bone formation at the center and margin of each defect is shown in Table 2.

**Table 2:** The mean percentage and standard deviation of NFB at the center and margin of defects.

	Mean %NFB (standard deviation)			
	4 weeks		8 weeks	
	Center	Margin	Center	Margin
Control	5.87 (7.05)	21.67 (1.79)	22.08 (13.20)	31.72 (6.23)
Bio-Oss	14.56 (5.29)	33.90 (5.42)	35.93 (8.60)	51.19 (6.11)
ANGIPARS	20.15 (8.96)	33.42 (4.15)	43.75 (6.57)	53.64 (6.31)
ANGIPARS + Bio-Oss	22.64 (9.54)	35.50 (5.65)	41.60 (11.58)	61.57 (7.65)

Although the most bone formation is observed in combination with ANGIPARS and Bio-Oss in the fourth week especially in center of the defect. ANGIPARS alone was more effective than Bio-OSS alone in both 4 and 8 weeks.

**Table 3:** The interaction effect of ANGIPARS and Bio-Oss on NFB.

			NFB in four weeks		NFB in eight weeks	
			Mean ± SD	*P *value	Mean ± SD	*P *value
(1)	Presence of Bio-Oss	Presence of ANGIPARS	31.34 ± 3.28^a^	0.005	54.93 ± 6.02	0.004
		Absence of ANGIPARS	26.44 ± 2.55		46.11 ± 4.20	
(2)	Absence of Bio-Oss	Presence of ANGIPARS	29.00 ± 2.31	<0.001	5.37 ± 5.36	<0.001
		Absence of ANGIPARS	16.30 ± 2.36		30.25 ± 2.43	
(3)	Presence of ANGIPARS	Presence of Bio-Oss	31.34 ± 3.28	0.12	54.93 ± 6.02	0.13
		Absence of Bio-Oss	29.00 ± 2.31		50.37 ± 5.36	
(4)	Absence of ANGIPARS	Presence of Bio-Oss	26.44 ± 2.55	<0.001	46.11 ± 4.20	<0.001
		Absence of Bio-Oss	16.30 ± 2.36		30.25 ± 2.43	

^a^As demonstrated, ANGIPARS has more synergic effect than BiO-Oss on NBF. *Row 1*. In presence of Bio-Oss, ANGIPARS showed a significant synergism effect on NBF both in 4 and 8 weeks even in the absence of Bio-Oss. *Row 2*. Even in absence of Bio-Oss, presence of ANGIPARS showed a significant effect on NBF in both 4 and 8 weeks. *Row 3*. In presence of ANGIPARS, Bio-Oss had no significant synergic effect on NBF in both 4 and 8 weeks. *Row 4*. In absence of ANGIPARS, Bio-Oss showed a significant effect on NBF in both 4 and 8 weeks.
